# A Case of Injury of the Knee-Joint with Successful Recovery

**Published:** 1855-07

**Authors:** William Watson


ARTICLE II.—*A Case of Injury of the Knee-Joint with Successful Recovery.* By William Watson, M. D.

March 23, was called to see T. B——, aged 10; found him with a transverse incision just above the patella of the right knee, about three inches in length. It commenced near the inner line of the tendon of the Triceps, extending downwards and outwards, having fractured the outer Tuberosity of the Tibia and severed the tendon of Triceps, laying open the knee-joint. By the contraction of the muscles the fractured bone was occupying the wound. The hemorrhage had been inconsiderable; having removed the coagula some synovia escaped from the wound. Upon replacing the fragment I found some difficulty in keeping it *in situ.* In order more effectually to do so and adapt the parts more accurately I placed two interrupted sutures in the divided tendon, taking care not to include more than one half the thickness of the tendon, having clipped one end close and brought the other out at the most dependent part. The wound was closed by interrupted sutures and adhesive straps, a roller being applied the whole length of the leg; it was placed on a long splint with the knee raised, (for this a fracture box was soon after substituted) and the whole was suspended so as to allow entire rest when the body was moved: cold water dressings were applied, and a dose of laxative medicine given to open the bowels.

24th and 25th. Saw the patient each day. No untoward symptoms have appeared; he has rested well and been entirely free from pain.

26th. Some inflammation and discharge, applied cold water freely.

27th. The inflammation has subsided, union by first intention having taken place to a considerable extent and the sutures in the integuments showing signs of ulceration they were removed; the tongue being coated and the bowels constipated. Gave pills of blue mass. and comp. ext. col.

28th to April 1st. The wound progressed favorably, no pain or irritation. The sutures of the tendon came away by slight traction on the 30th, (the 8th day.)

2d. Found the patient, having indulged too freely in amusement the previous day, knee swollen and painful, applied cold water freely with diaphoretics internally. The fever assuming an intermittent type yielded readily to antiperiodics.

6th. Saw the patient daily, he had been improving slowly, but to-day found it much swollen with a pulpy appearance of the wound; covered it with sub. mur. hydg., applied dry lint and firm compression to the upper part of the wound, by means of adhesive straps running lengthwise of the leg.

7th. The appearance of the knee much improved, the inflammation having subsided considerably. But the swelling remaining, and there being but little discharge a linen tent was introduced into the wound and the dressings continued.

8th. There has been considerable discharge, the wound looking healthy, from this time until the 14th it continued to improve, when the discharge became interrupted with a tendency of the matter to burrow among the muscles of the thigh and about the joint. The roller was reapplied firmly and readjusted as often as it became loosened, or there was any tendency to swelling, until the 25th, when it being nearly healed all dressings were removed except the roller which was continued some weeks to counteract the tendency to swelling of the joint.

The patient was discharged at the expiration of the fifth week, with the knee entirely healed and sufficient motion to allow about one half the usual extent of flexion without pain.

At this time, eleven weeks since the injury, the joint has recovered nearly the natural motion, and he walks with a scarcely perceptible limp.

